# Implementation of scientific evidence into rheumatological care—the viewpoint of the EULAR

**DOI:** 10.1016/j.ero.2026.100265

**Published:** 2026-11-09

**Authors:** George S. Metsios, George E. Fragoulis, Bernhard B. Hellmich, Alessandro Consolaro, Bruno Fautrel, Jet JCS Veldhuijzen van Zanten, Annette Helena Maria van der Helm van Mil, Loreto Carmona, Krysia S. Dziedzic, Dirk Foell, Anastasia-Vasiliki Madenidou, Simon R. Stones, Peter Boyd, Souzi Makri, Alessandro Tomelleri, Uta Kiltz

**Affiliations:** 1 School of Physical Education, Sport Science and Dietetics, Department of Nutrition and Dietetics, University of Thessaly, Trikala, Greece; 2 First Department of Propedeutic and Internal Medicine, National and Kapodistrian University of Greece, Athens, Greece; 3 Medius Kliniken, Teaching Hospital of the University of Tubingen, Kirchheim-Teck, Germany; 4 Pediatric Rheumatology and Autoinflammatory Diseases Unit, Istituto Giannina Gaslini, Genova, and Department of Neurosciences, Rehabilitation, Ophthalmology, Genetics, and Maternal-Child Sciences (DiNOGMI), University of Genoa, Genoa, Italy; 5 Sorbonne University – Assistance Publique Hôpitaux de Paris (APHP), Institut National de la Santé et de la Recherche Médicale (INSERM), Unité Mixte de Recherche Scientifique (UMRS) 1136, CRI-IMIDIATE (Club Rhumatismes et Inflammation – Immune-Mediated Inflammatory Disease Alliance for Translational and Clinical Research) network, CEREMAIA (Centre de Référence pour les Maladies Auto-Inflammatoires et les Amy loses) Reference Center, Department of Rheumatology, Pitie-Salpetriere Hospital, Paris, France; 6 Centre of Movement and Wellbeing, School of Sport, Exercise and Rehabilitation Sciences, University of Birmingham, UK; 7 Department of Rheumatology, Leiden University Medical Center, Leiden, the Netherlands; 8 Instituto de Salud Musculoesquelética (INMUSC), Madrid, Spain; 9 Impact Accelerator Unit, School of Medicine, Faculty of Medicine and Health Sciences, Keele University, Staffordshire, UK; 10 Department of Paediatric Rheumatology and Immunology, University of Muenster, Muenster, Germany; 11 Centre for Musculoskeletal Research, Division of Musculoskeletal and Dermatological Sciences, The University of Manchester, Manchester, UK; 12 University of Salford, Salford, UK; 13 Amica Scientific, Macclesfield, UK; 14 Patient Expert, Founder of Patient Bridge, Dublin, Ireland; 15 Cyprus League of People with Rheumatism, Nicosia, Cyprus; 16 Unit of Immunology, Rheumatology, Allergy and Rare Diseases, Istituto di Ricovero e Cura a Carattere Scientifico (IRCCS) San Raffaele Hospital, Milan, Italy; 17 Ruhr-University Bochum, Bochum, Germany; 18 Rheumazentrum Ruhrgebiet, Herne, Germany

## Abstract

The gap between scientific discoveries and bedside application remains one of the most significant challenges in modern healthcare. Implementation science can aid this process by ensuring that health practices evolve alongside scientific progress rather than lagging years behind. This viewpoint focuses on the efforts of the European Alliance of Associations for Rheumatology to foster the implementation process of recommendations for rheumatic and musculoskeletal disease by addressing implementation strategies and by outlining barriers and facilitators for supporting interdisciplinary and patient-centred care.

## BACKGROUND

The gap between scientific discoveries and bedside application remains one of the most considerable challenges in modern healthcare [1,2]. Although scientific research continuously generates innovative treatments and diagnostic tools, their potential is only realised when these are effectively integrated into routine clinical workflow [3]. A lack of translating research findings into routine clinical practice exists even for cumulative evidence that is summarised into therapeutic and diagnostic recommendations. Implementation science has become integral in ensuring that scientific progress directly informs and advances clinical practice. To effectively bridge the evidence-to-practice gap, research organisations that provide funding, including the European Alliance of Associations for Rheumatology (EULAR), now require researchers to propose implementation strategies to ensure that research investment creates impact for the target population, namely people living with rheumatic and musculoskeletal diseases (RMDs). Among many other tasks, EULAR, as a leading nonprofit European organisation, not only plays a leading role in developing recommendations for better managing RMDs but also provides funding opportunities for researchers and practitioners to advance the translation of research into practice. Given the importance of its published recommendations, the EULAR focuses on the use of implementation science methodologies to support and enhance the uptake of EULAR recommendations. The aim of the present paper, therefore, is (i) to highlight the implementation challenges identified in recent years and (ii) to provide an update on the advances that EULAR has recently focused on to promote implementation science and thus enable translation of recommendations in routine clinical practice.

## USING IMPLEMENTATION SCIENCE TO IMPROVE PATIENT OUTCOMES

The translation of scientific findings into actionable clinical practice (such as the EULAR recommendations) not only provides standardised guidance for professional conduct but also, importantly, improves patient health as well as the quality of healthcare services. For example, adhering to quality guidelines can not only prevent significant comorbidities and reduce mortality but also significantly reduce healthcare costs [4]. A significant challenge, however, is how high-quality guidelines and recommendations can be adopted in routine clinical practice, given that there is an average delay of >10 years from publication of scientific literature and its application within healthcare systems [5,6]. At present, and despite the increasing number of research studies, <15% of research evidence translates into actionable practice within healthcare services, suggesting a concerningly low implementation rate [7].

Implementation science has been developed since 2000 to promote the knowledge translation and systematic uptake of effective evidence-based practices into routine care. Although clinical research focuses on whether an intervention works (efficacy), applied health research focuses on whether it works in practice (effectiveness) and knowledge mobilisation investigates the why, whose, what, and how [8]. This is why the science of implementation is supported by process models and evaluation frameworks. Across European countries, there are significant contextual and operational differences between healthcare systems. However, implementation science provides a framework for adopting effective healthcare interventions that are tailored to these specificities, provided that a systematic approach is adopted (Fig 1).

**Figure 1:**
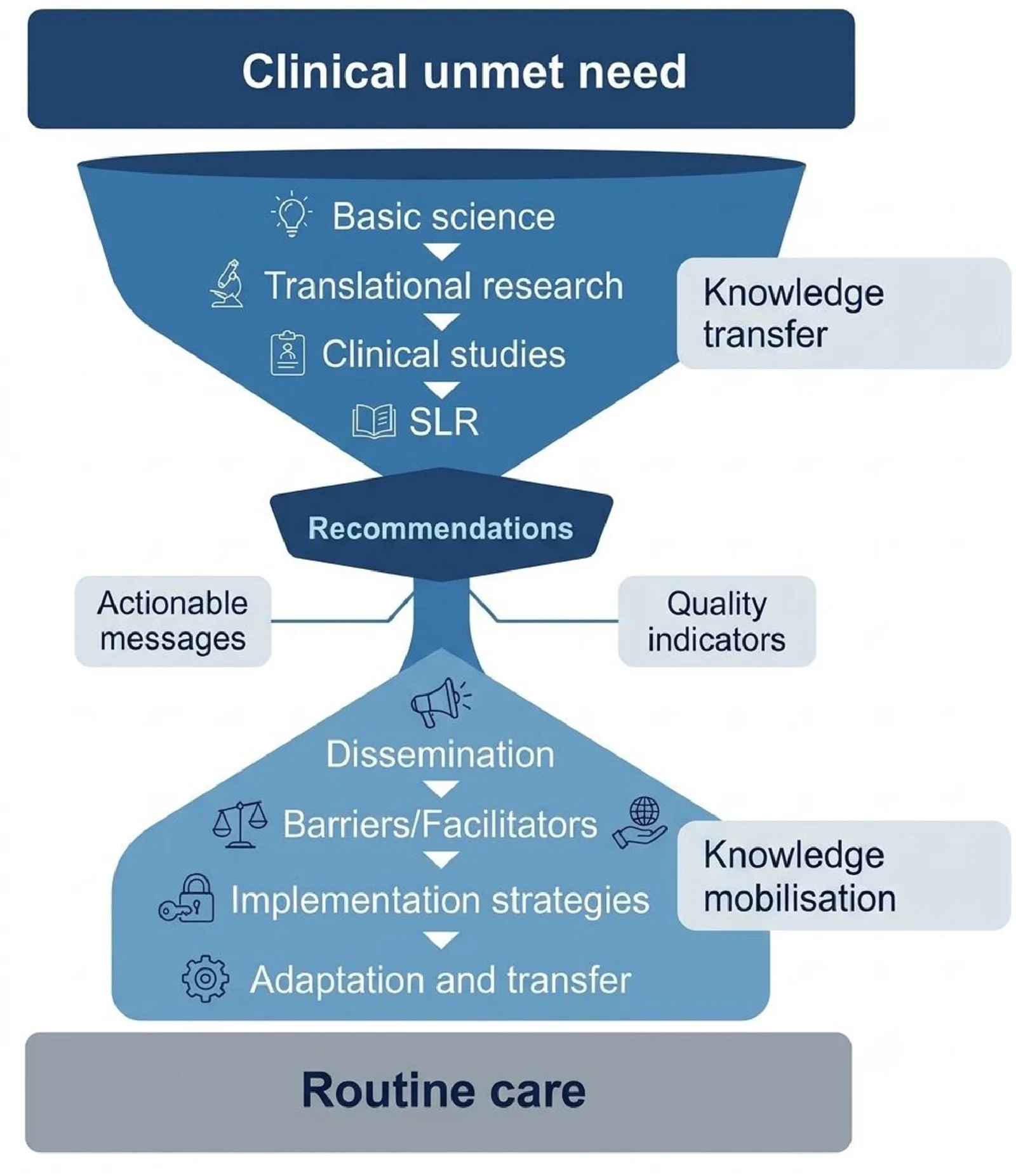
Graphical representation of implementation.

## IMPLEMENTATION CHALLENGES

One of the key roles of EULAR is to produce clear recommendations on the diagnosis and multidisciplinary management of RMDs, based on the current state of the art. To achieve this, specific conditions need to be satisfied: (i) clear formulation of recommendations, (ii) identification of key quality indicators to measure the extent to which EULAR recommendations have an impact on RMD patient care, and (iii) provision of a set of educational tools targeting different stakeholders (patients, rheumatologists, paediatric rheumatologists, and healthcare practitioners) that can be used at the national level to facilitate implementation. However, significant challenges still remain at the micro (patient and health care professionals), meso (healthcare organisations and systems) and macro (national policy) levels.

### Challenges related to patient care

Barriers to the uptake of evidence-based recommendations do exist, despite the high quality of process and robust methodology that is required to develop the EULAR recommendations. Specifically, low evidence literacy in clinicians and inaccessibility of information in populations with low health literacy may limit optimal engagement with EULAR recommendations. The fact that EULAR recommendations are published in English has also been highlighted as a potential barrier to dissemination and implementation. Moreover, financial barriers (eg, multilevel and multistakeholder capacity building) combined with the complex care for multimorbidity management in RMDs may also inhibit effective translation of research into practice and evolving healthcare pathways. Patient-focused facilitators, peer support, digital tools, and inclusive patient dissemination channels, as well as plain language summaries that are engaging and accessible, may be some of the potential facilitators across European countries.

### Heterogeneity of healthcare systems across Europe

The EULAR mission is not to deliver the implementation of its recommendations at the national level since specific stakeholders may consider such actions not applicable to their healthcare systems (for different reasons including context, lack of funding and human resources). Instead, EULAR actions are dedicated to the provision of optimal and accurate information to the large community of rheumatologists, paediatric rheumatologists, and healthcare professionals, people with RMDs and related stakeholders. European countries have different healthcare protocols and access to specific medication and behavioural intervention pathways [9]. Therefore, publishing clear and evidence-based recommendations can help European stakeholders prioritise which recommendations can be adapted and tailor them to their own system and context for improving patient care. Collaboration of EULAR with National Societies may also help not only to understand potential barriers at the national level but also to disseminate more effectively the EULΑR recommendations to the end users, thus enhancing the possibility of implementation.

### Resistance to change

Resistance to changing routine care is both a human and a societal challenge, far beyond EULAR recommendations. Even though EULAR is publishing—through robust and high-quality methodologies—evidence-based, up-to-date and clear recommendations, resistance to adopting these remains a significant challenge. Barriers to changing healthcare pathways are complex, multifunctional and multifactorial, and are influenced at different levels (micro, meso, macro) by various factors inside and outside a healthcare system. This suggests that healthcare strategies involving new recommendations need to reflect the multidimensional nature of quality of care and be incorporated at different levels, thus tailoring interventions to end-user needs. For this reason, and through targeted strategies that recognise these matters, the EULAR mission is to provide relevant and actionable messages to improve RMD care as well as operational tools to facilitate knowledge mobilisation and implementation of recommendations into daily clinical practice.

## EULAR TARGETED STRATEGIES FOR ENHANCING IMPLEMENTATION

Over the past decade, EULAR has endorsed a significant number of recommendations. However, as previously mentioned, their implementation in daily rheumatological practice remains a significant issue. For example, studies have focused on understanding potential barriers and facilitators for the uptake of the EULAR recommendations for patient education from both a healthcare professional and patient perspective [10,11]. These studies, conducted across 23 European and Asian countries, demonstrated that there is a high level of agreement for implementing the EULAR recommendations; however, their application in clinical practice was low due to a lack of time and personnel, as well as a lack of training and evaluation tools. These barriers have also been identified in other EULAR recommendations [12]. To enhance implementation of EULAR recommendations, EULAR has strategically undertaken a series of actions: (i) funding implementation science projects, (ii) adopting standard operating procedures (SOPs) to integrate implementation criteria into the development of new recommendations, and (iii) providing guidance describing the different steps required to facilitate and optimise implementation at national level [13]. An overview of the EULAR strategic plan appears in Figure 2.

**Figure 2:**
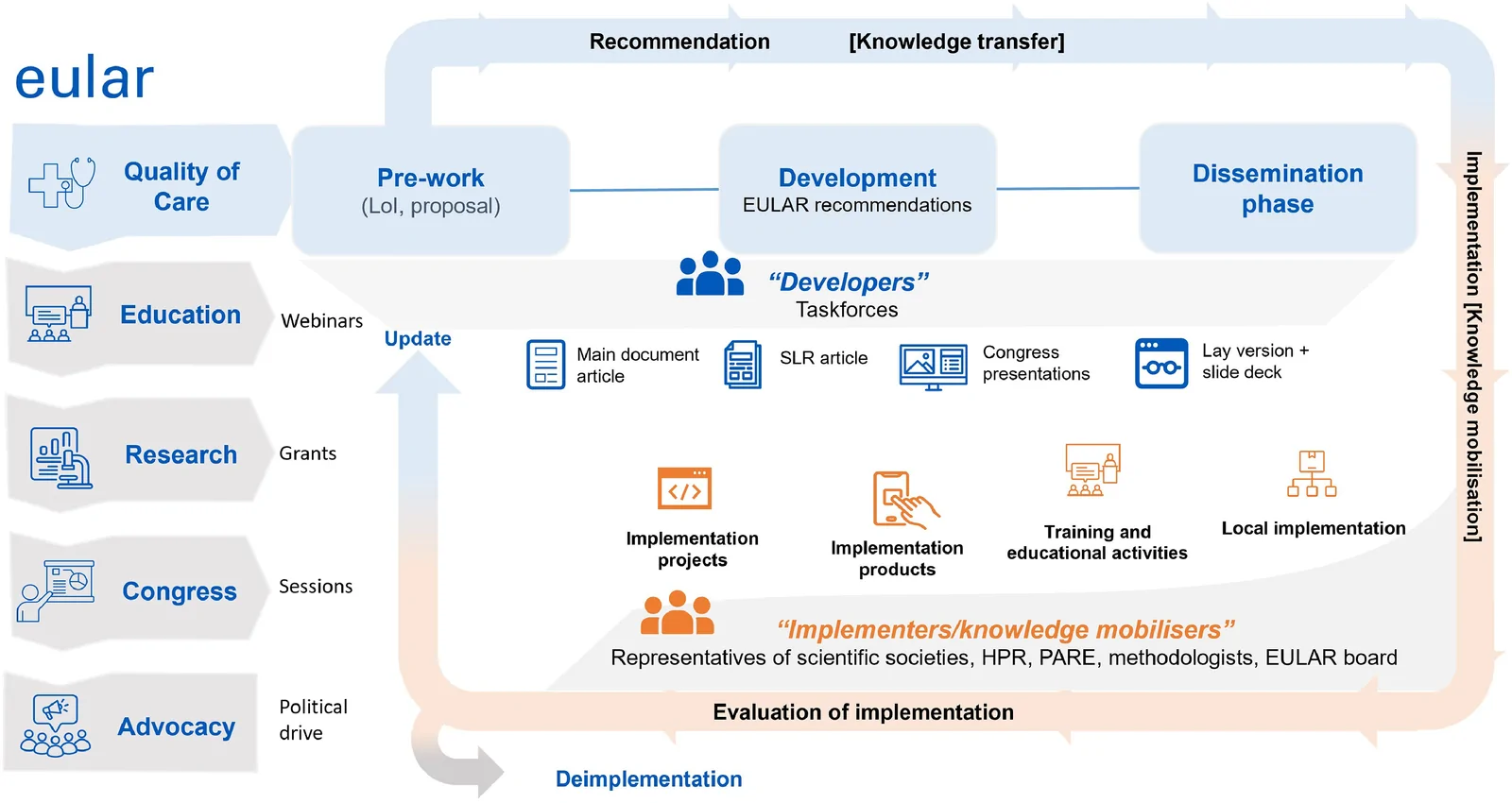
European Alliance of Associations for Rheumatology (EULAR) strategic actions to enhance implementation. Figure 2 represents the support for the implementation life cycle across all EULAR content committees. Quality of Care is the leading committee for the implementation plan. It is also responsible for prioritising and coordinating the development of EULAR recommendations. In this regard, the prework for recommendations has changed (Letter of intent/proposal for EULAR recommendations) and should now include implementation evidence in updates, context analysis, and a dissemination and knowledge mobilisation plan (at EULAR and national level, scenarios, deliverables). The standard operating procedures (SOPs) for developing EULAR recommendations include the ‘perspective on implementation’, ie, the wording of recommendations, prioritisation of core recommendations, context and barriers, and implementation data if available. Also, in the dissemination phase, the SOPs recommend considering implementation objectives, context, barriers, and facilitators, and using practical tools, such as algorithms or checklists, depending on the knowledge mobilisation needs. The EULAR Education Committee supports implementation by holding educational and training activities on implementation and knowledge mobilisation. The Research Committee supports implementation through grants for implementation and knowledge mobilisation research projects. The Congress Committee is also involved by including implementation sessions and keywords in the programme. The EULAR Study Group on Implementation, which acts as an observatory for the whole plan and coordination and stems from Quality of Care, holds its annual on-site meeting during the Congress. Finally, the Advocacy Committee supports the implementation plan by aligning stakeholders and providing advice, and by planting the seed of the political drive.

## EULAR POST-TASKFORCE IMPLEMENTATION FUNDING

The recent attempts by EULAR to enhance the implementation of its recommendations have also expanded into integrating implementation aspects into the SOPs for TaskForces, ie, the groups of experts that develop the EULAR recommendations. Specifically, the TaskForce composition, which includes patients and healthcare professionals from different countries and with diverse backgrounds, promotes a more effective approach at macro, meso, and micro levels, given that the translation of research into clinical care requires input from all those key stakeholders that can influence its efficacy. Moreover, EULAR now requires TaskForces to provide implementation strategies and quality indicators when developing recommendations.

Importantly, EULAR now provides funding support via specific Post-TaskForce implementation projects. This initiative ensures that the work of EULAR TaskForces is not concluded with a peer-reviewed publication, but continues through actionable, evidence-based strategies to ensure consistent, high quality, and sustainable translation of guidelines into clinical practice. These SOPs are not static documents but dynamic tools that guide the entire implementation lifecycle: project planning, key stakeholder engagement, needs assessment, implementation strategy development, monitoring, and evaluation, as well as sustainability. Moreover, the involvement of all key stakeholders in this process, namely rheumatologists, healthcare practitioners, patient organisations, national societies, and healthcare providers, further enhances the possibility for effective implementation of EULAR recommendations. Involving policymakers and health authorities for the management and treatment of RMDs further offers a major strategic advantage: their engagement helps ensure that evidence-based recommendations are translated into coordinated health policies, adequate resource allocation, and consistent standards of care across regions.

## ADVOCACY AND COLLABORATION WITH COMMUNITIES OF PRACTICE

EULAR advocacy is crucial for implementing recommendations. EULAR engages with the EU and political institutions at the national level and advocates research and education to make the needs of RMD patients recognisable. Through the publication of high-quality recommendations, EULAR provides strategic guidance as a source of expertise in RMDs, bringing RMDs onto the EU’s agenda. In addition, EULAR People with Arthritis/Rheumatism in Europe collaborates with different communities of practice across different nations through interdisciplinary Rheumatology Societies and with public involvement to enhance the adoption of EULAR recommendations. This is an important bottom-up implementation approach that relies on social innovation. These strategies are well placed to develop relationships, trust, clinical champions, knowledge brokers, and cultural adaptation, all of which accelerate the uptake of innovation. These EULAR communities offer context-specific innovations, resources, experience with clinical audit, project management, and case studies, all delivered with flexibility aligned not only with their national frameworks but also with the EULAR recommendations.

## CONCLUSION

EULAR has the potential to become a leader in the implementation of quality care for RMDs, and this can be achieved via an evidence-based, scalable, and sustainable framework. Embedding implementation science methods and frameworks into recommendation development, and supported by relevant funding, can help maximise the likelihood of success in improving the quality of healthcare and healthcare services for RMDs, in a sustainable and scalable manner.

## CRediT authorship contribution statement

**George S. Metsios:** Writing – original draft, Visualization, Supervision, Resources, Methodology. **George E. Fragoulis:** Writing – review & editing. **Bernhard B. Hellmich:** Writing – review & editing. **Alessandro Consolaro:** Writing – review & editing. **Bruno Fautrel:** Writing – review & editing. **Jet JCS Veldhuijzen van Zanten:** Writing – review & editing. **Annette Helena Maria van der Helm van Mil:** Writing – review & editing. **Loreto Carmona:** Writing – review & editing. **Krysia S. Dziedzic:** Writing – review & editing. **Dirk Foell:** Writing – review & editing. **Anastasia-Vasiliki Madenidou:** Writing – review & editing. **Simon R. Stones:** Writing – review & editing. **Peter Boyd:** Writing – review & editing. **Souzi Makri:** Writing – review & editing. **Alessandro Tomelleri:** Writing – review & editing. **Uta Kiltz:** Writing – original draft, Supervision, Project administration, Conceptualization.

## Competing interests

UK reports financial support from AbbVie, Alfasigma, Amgen, Biocad, Biogen, Chugai, Eli Lilly, Fresenius, GBA, Gilead, Grünenthal, GSK, Hexal, J&J, MSD, Novartis, Pfizer, Roche, Sanofi, and UCB. UK has received board membership fees, consulting or advisory fees, grants, speaking and lecture fees, and travel reimbursement from these organisations. UK is a board member of Zeitschrift für Rheumatologie and past chair of the European Alliance of Associations for Rheumatology (EULAR) Quality of Care Committee. BBH is a member of the EULAR Quality of Care Committee. KSD reports administrative support from the EULAR. KSD has received board membership fees, grants, speaking and lecture fees, and travel reimbursement from EULAR, the National Institute for Health and Care Research, the National Institute for Health and Care Excellence, Health Innovation West Midlands, Birmingham Biomedical Research Centre, and Arthritis UK. KSD is chair of the EULAR Implementation Study Group. AVM has received grants from the NIHR Manchester Biomedical Research Centre and Johnson & Johnson Pharmaceutical Research & Development LLC and speaking and lecture fees from Boehringer Ingelheim Ltd. SRS reports administrative support from EULAR and has received board membership fees, nonfinancial support, and travel reimbursement from EULAR. SRS is employed by Amica Scientific Ltd and has received employment compensation and equity or stock from Envision Pharma Group Limited. SRS has received consulting or advisory fees and travel reimbursement from the National Institute for Health and Care Research and The Kennedy Trust for Rheumatology Research; consulting or advisory fees from Sage Publications Ltd, Taylor & Francis Group Limited, and the University of Leeds; and board membership fees, consulting or advisory fees, and speaking and lecture fees from the University of British Columbia. SRS is also a board member of Raising Awareness of Invisible Illnesses in Schools and Education (RAiISE) and the International Society for Medical Publication Professionals.

AT has received board membership fees and speaking and lecture fees from AbbVie and Novartis and speaking and lecture fees from Alfasigma, Eli Lilly & Co, Fresenius Kabi, Johnson & Johnson, and UCB. The other authors declare no known competing financial interests or personal relationships that could have appeared to influence the work reported in this paper.
